# Allicin Attenuates Inflammation and Suppresses HLA-B27 Protein Expression in Ankylosing Spondylitis Mice

**DOI:** 10.1155/2013/171573

**Published:** 2013-11-13

**Authors:** Xin Gu, Haishan Wu, Peiliang Fu

**Affiliations:** ^1^Department of Orthopaedics, Shanghai Tenth People's Hospital Affiliated to TongJi University, Shanghai 20072, China; ^2^Department of Orthopaedics, The Second Affiliated Hospital of the Second Military Medical University, No. 415 Fengyang Road, Huangpu District, Shanghai 200003, China

## Abstract

Here we aimed to determine the therapeutic effect of allicin on ankylosing spondylitis (AS) and explore the mechanism(s) of action. AS mouse model was constructed by transferring the HLA-B2704 gene into Kunming mice and verified by RT-PCR and CT imaging. Verified AS mice were randomly divided into model group (*n* = 6) and allicin-treated groups (50, 100, and 200 mg/kg, resp., *n* = 6, p.o., for 2 months). Wild type mice were used as control (*n* = 6). The levels of AS-related inflammatory factors were measured by ELISA. mRNA and protein expressions of HLA-B27 were checked by RT-PCR and western blotting. As the results, the mouse model of AS was successfully established, and high-dose allicin could markedly alleviate spine inflammatory injury possibly via reducing the secretion of the inflammatory factors (IL-6, IL-8, and TNF-**α**) sharply in AS mice. Moreover, allicin significantly inhibited HLA-B27 protein translation but failed to suppress HLA-B27 gene transcription in AS mice, indicating a posttranscriptional mechanism of this modulation. In conclusion, allicin has potential to be used for AS treatment as an anti-inflammatory nutraceutical.

## 1. Introduction

Ankylosing spondylitis (AS), a chronic inflammation disorder, occurs in lumbar spine and sacroiliac peripheral joints. The type and progression of AS are associated with prognostic factors such as race, gender, age at onset, and early axial skeleton involvement [1]. The incidence of AS among xanthoderm is about 2‰  and males are more easily affected than females [2]. Pathogenesis of AS is strongly related to major histocompatibility complex (MHC) class I, and about 95% of patients with AS are born to have the HLA-B27 gene, which leads to a tendency for familial genetic association [3, 4]. However, the mechanism of AS onset is still unclear, and effective therapies are urgently needed for this disease. It was reported that the levels of inflammatory factors increased in AS patient, including TNF-*α*, IL-6, and IL-10 [4, 5]. Inhibiting the release of these inflammatory factors could partly ease the symptoms of AS and appears to reduce disease severity as well.

Garlic (*Allium sativum L.*) was considered as a food with many therapeutic activities including antioxidant, antibacterial, and anticholesterol activities [6–11]. Allicin, as one of the metabolites of garlic, is responsible for most of the functions of garlic [12]. Researches revealed that allicin could have different biological activities such as antibacterial effects [13, 14]. Moreover, allicin can reduce the release of TNF-*α* and IL-8, and has the function of immune regulation [7], indicating that it may have potential to treat systemic autoimmune disease (e.g., AS).

In this study, we aimed to investigate whether allicin has a therapeutic effect on AS. We established AS model in HLA-B2704 (a common subtype of HLA-B27) transgenic mice, treated them with allicin, and measured relevant inflammatory factors as well as HLA-B2704 mRNA and protein levels in order to find out a promising therapeutic approach for AS.

## 2. Materials and Methods

### 2.1. Materials and Chemicals

Allicin chloride was obtained from Changsha Yaying Bio-tech Co.,  Ltd. (Changsha, China). Trizol was purchased from Invitrogen Corporation (Carlsbad, CA, USA). ECL kit and skimmed milk powder were purchased from Guangzhou Maygene Biotech Co., Ltd. (Guangzhou, China). Protein extraction kit was purchased from Shanghai Times Bio-tech Co., Ltd. (Shanghai, China).

### 2.2. Construction of AS Mouse Model and Drug Administration

All animal studies have been approved by China Ethics Committee and performed in accordance with the ethical standards. Kunming mice were obtained from the Laboratory Animal Institute of Chinese Academy of Medical Sciences. Plasmid (pBR322-HLA-B2704) was provided by our laboratory. HLA-B2704 DNA fragment was prepared via amplification, EcoR I digestion, electrophoresis, extraction, and purification. AS model mice were constructed by transgene method [15]. Three hundred mice were injected with hormone for super ovulation. Zygotes were collected and HLA-B2704 gene DNA fragment was injected into pronucleus by microinjection. Survived zygotes of second cell stage were transferred into pseudocyesis mice for generation.

The AS mice were randomly divided into AS model group (*n* = 6) and allicin-treated groups. Treatment dosages were 50, 100, and 200 mg/kg body weight [16] for the low-dose group (*n* = 6), the moderate dose group (*n* = 6), and the high-dose group (*n* = 6), respectively. The drug was infused to stomach of mice every morning, whereas the control group (wild type Kunming mice, *n* = 6) and the model group received no addition. All the mice were killed after 2 months of treatment and tissue sample near foot joint as well as peripheral blood from individual animals was collected.

### 2.3. Reverse Transcription-Polymerase Chain Reaction (RT-PCR) Assay

Total RNA (from peripheral blood) was prepared by the acid phenol method using Trizol reagent as instructed by the manufacturer. RT-PCR was performed as described previously, and the reverse-transcribed cDNA was then amplified by PCR using the following primers: HLA-B27, 5′-GGGTCTCACACCCTCCAGAAT-3′ (sense), and 5′-CGGCGGTCCAGGAGCT-3′ (antisense). PCR conditions were as follows: one cycle of 48°C for 45 min and 96°C for 2 min; 95°C for 30 s, 60°C for 45 s, and 72°C for 30 s, for 40 cycles; the last cycle for 7 min at 70°C. PCR products were separated by electrophoresis on 2% agarose gels and visualized by staining with ethidium bromide. The sequence length of target product is 135 bp.

### 2.4. CT Imaging

Siemens 16 layers spiral CT scanner was used for imaging. The parameters were as follows: rotational speed 0.4 r/s, precision diameter 0.75 mm, tube voltage 120 kV, tube current “automatic mA control,” screw pitch 1 mm, and scanning layer thickness 0.75 mm for 16 layers.

### 2.5. Real-Time Quantitative PCR

Total RNA (from tissue sample near foot joint) was prepared as mentioned above. HLA-B27 primer sequences were designed by an online tool (http://www.idtdna.com/), and *β*-actin primer sequences were obtained from the reference [17]. The primer sequences are as follows: HLA-B27, 5′-GAGAACGGGAAGGACAAGC-3′ (forward), 5′-GATCTCCGCAGGGTAGAAAC-3′ (reverse); *β*-actin, 5′-AGCGGGAAATCGTGCGTGAC-3′ (forward), 5′-ACTCCTGCTTGCTGATCCACATC-3′ (reverse). mRNA level was measured by SYBR green real-time PCR assay. HLA-B27 gene amplification was carried out as follows: reverse transcription at 48°C for 45 min; initial activation of HotStar Taq DNA Polymerase at 96°C for 2 min; 40 cycles in three steps: 95°C for 30 s, 60 °C for 30 s, and 72°C for 30 s. Data acquisition and analysis were carried out using CFX-96 real-time quantitative PCR instrument (Bio-Rad) and SPSS 15.0 software.

### 2.6. Enzyme-Linked Immunosorbent Assay (ELISA)

TNF-*α*, IL-6, and IL-8 secretion was analyzed by sandwich ELISA. Antibodies (rabbit anti-TNF-*α*, anti-IL-6, and anti-IL-8 antibody) were added to 96-well plates and incubated overnight at 4°C. The plates were then washed and blocked with 2% BSA. Samples and corresponding standard samples were added to the wells and the plate was incubated at 4°C for 30 min. After washing, the wells were incubated with a biotinylated goat anti-rabbit secondary antibody and peroxidase labelled avidin at 4°C for 30 min. Following multiple washes, chromogenic agent was added into the wells and the optical absorbance was detected at 405 nm. Concentration of samples was calculated according to the standard curve.

### 2.7. Western Blotting Analysis

HLA-B27 and *β*-actin expression were determined by western blotting analysis. Whole proteins were prepared by protein extraction kit. Equal amounts of proteins (20 *μ*g) were separated by 12.5% SDS-PAGE and transferred to PVDF membranes, which were blocked with 5% skimmed milk and incubated with the rabbit anti-HLA-B2704 antibody and anti-*β*-actin antibody. These were followed by incubation with the goat anti-rabbit antibody conjugated with horseradish peroxidase as the secondary antibody for 1 h. Immunoreactive bands were detected by X-ray film developer.

### 2.8. Statistical Analysis

The data were expressed as mean ± SEM and analyzed by one-way ANOVA or two-tailed unpaired Student's *t*-test. A probability value of less than 0.05 was considered statistically significant. All results are representative of at least three independent experiments.

## 3. Results

### 3.1. Construction of AS Mouse Model

**Figure 1:**
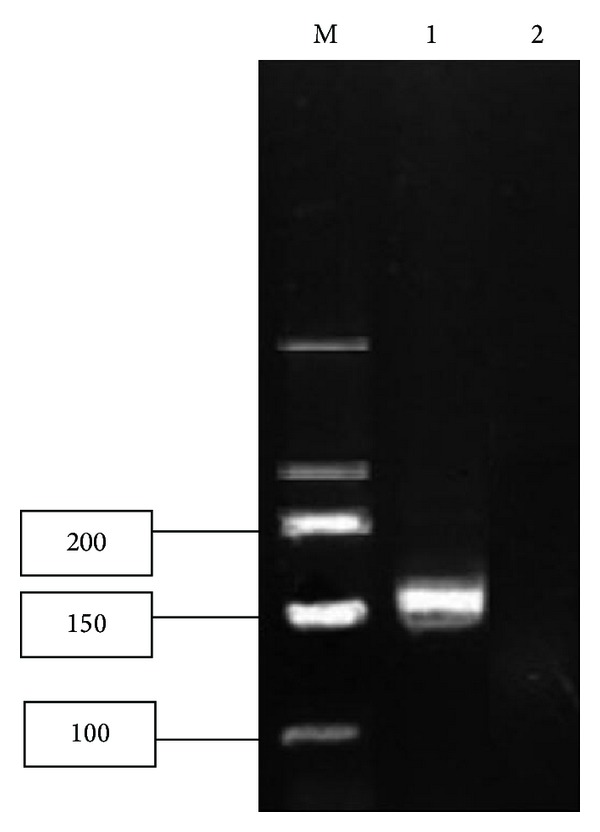
Verification of the expression of HLA-B2704 in peripheral blood by RT-PCR. M: marker; 1: positive group; 2: control group.

HLA-B2704 gene expression in peripheral blood of the AS mice was measured by RT-PCR (Figure 1). A total of 26 positive AS model mice were obtained, in which HLA-B2704 gene was expressed.

**Figure 2:**
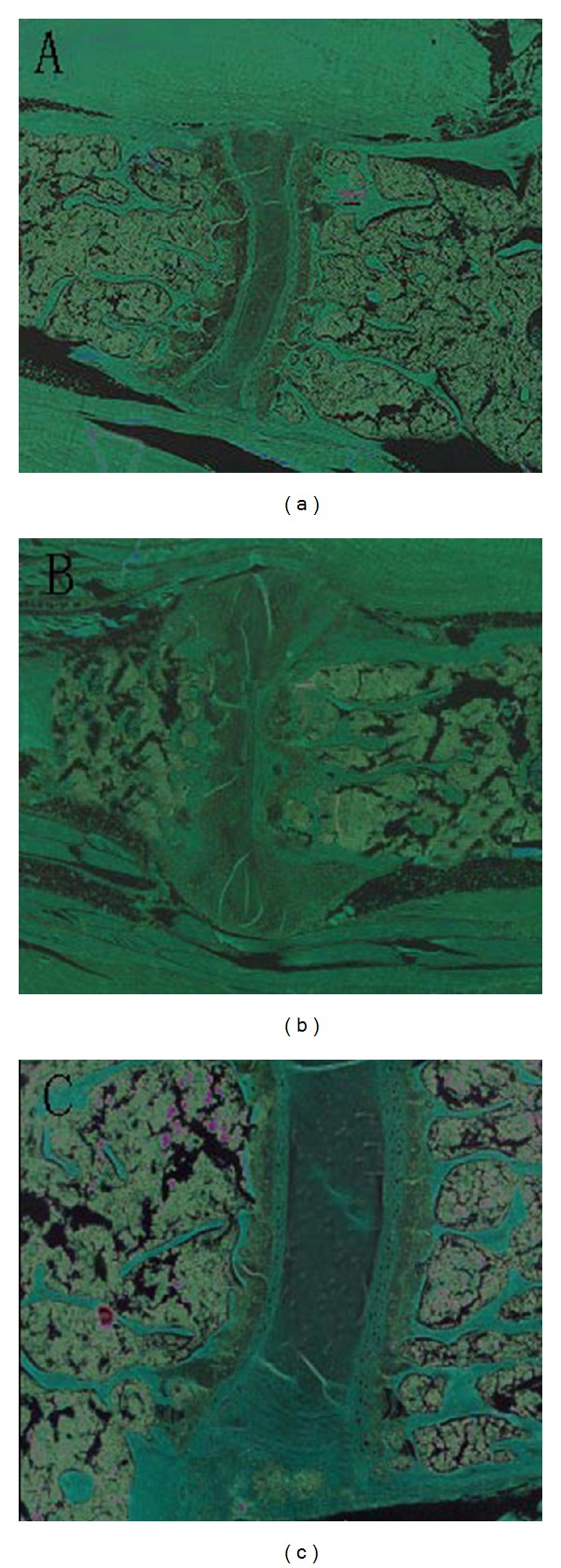
Effects of allicin on spine impairment of AS mice. Siemens 16 layers spiral CT scanner was used for imaging. (a) Control mice; (b) AS model mice; (c) 200 mg/kg allicin-treated AS mice.

It was found by CT imaging that the spine of control mice was normal, as articular surface was smooth and intact, and spine interval has no deformation (Figure 2(a)). However, the spine interval of AS model mice was changed obviously, as the continuity was lost (Figure 2(b)). 

### 3.2. Effects of Allicin on Symptoms of AS Mice 

Mice of control group showed no indisposition phenomenon. Model group showed severe red swelling in foot and toes and local depilation. After treated with 50 mg/kg or 100 mg/kg of allicin, red swelling and depilation were alleviated. The group which received 200 mg/kg allicin showed no apparent red swelling and depilation phenomenon in foot and skin, and the impaired spine was almost recovered (Figure 2(c)).

### 3.3. Effects of Allicin on the Release of Inflammatory Factors

**Figure 3:**
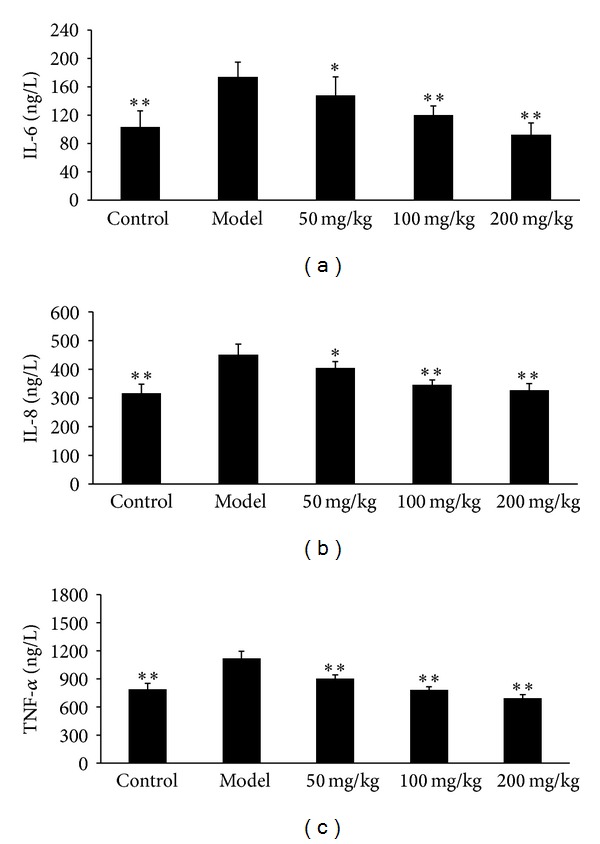
Effects of allicin on the releasing of IL-6, IL-8, and TNF-*α* in HLA-B2704 transgenic mice. Mice were orally administered with allicin (50 mg/kg, 100 mg/kg, and 200 mg/kg, resp.) every morning for 2 months. IL-6, IL-8, and TNF-*α* were tested by ELISA analysis. All data presented are the mean ± SEM of 6 mice. (a) IL-6; (b) IL-8; (c) TNF-*α*. *P < 0.05, **P < 0.01 versus model group.

As illustrated in Figure 3, the release of IL-6, IL-8, and TNF-*α* was significantly increased in AS model mice compared with control mice, indicating that there were inflammation reactions in joints. Allicin could extremely inhibit these three inflammatory factors releasing in a dose-dependent manner. Allicin at the dose of 100 mg/kg could reduce the level of inflammatory factors to normal, and 200 mg/kg allicin even reduced IL-6 and TNF-*α* to the levels a little lower than normal (Figures 3(a) and 3(c)).

### 3.4. Effects of Allicin on mRNA Content of HLA-B27 in AS Mice

**Figure 4:**
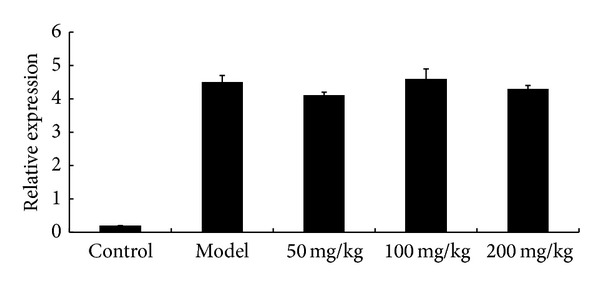
Effects of allicin on HLA-B2704 gene expression. Mice were orally administered with allicin (50 mg/kg, 100 mg/kg and 200 mg/kg, resp.) every morning for 2 months. HLA-B2704 mRNA expression was analyzed by Real-time fluorescence quantitative PCR analysis. All data presented are the mean ± SEM of 6 mice. **P < 0.01 versus Model group.

HLA-B2704 mRNA level in joints was determined by fluorescence quantitative PCR. As shown in Figure 4, mRNA level of HLA-B27 in AS model mice was not altered significantly by allicin treatment, indicating that allicin might not affect the transcription of HLA-B2704 gene.

### 3.5. Effects of Allicin on HLA-B2704 Protein Expression in AS Mice

**Figure 5:**
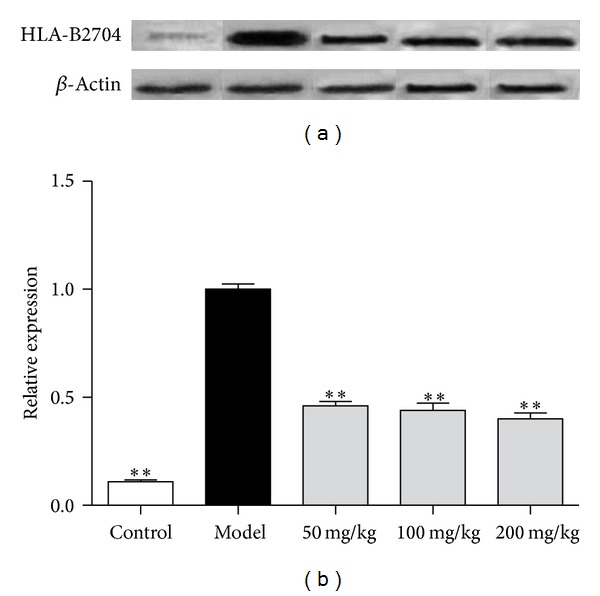
Effects of allicin on HLA-B2704 protein expression. Mice were orally administered with allicin (50 mg/kg, 100 mg/kg, and 200 mg/kg, resp.) every morning for 2 months. HLA-B2704 expression was analyzed by western blotting analysis. (a) Blots of HLA-B2704 and *β*-actin; (b) quantitative analysis of the blots. All data presented are the mean ± SEM of 6 mice. **P < 0.01 versus model group.

HLA-B2704 protein expression was investigated by western blotting analysis. Results showed that HLA-B27 protein expression was significantly increased in AS model group. Allicin showed an inhibiting effect on HLA-B2704 protein expression in all three doses, indicating that allicin could inhibit HLA-B2704 translation (Figure 5).

## 4. Discussion

This study investigated the effects of allicin on AS in HLA-B2704 transgenic mice. Our results show that allicin could ameliorate spine impairment and reduce the release of IL-6, IL-8, and TNF-*α* in AS model mice. Specifically, we found out that allicin could inhibit HLA-B2704 protein expression but had no effect on the expression of HLA-B2704 gene, suggesting a posttranscriptional mechanism of the modulation of allicin on HLA-B2704 gene.

Researches have shown that allicin could affect immune function in mice [18, 19]; that is, allicin increases the weight of the spleen and thymus, activates T-lymphocyte, and stimulates the secretion of mononuclear cell and lysozyme releasing. Thus, allicin could improve cellular immunity, humoral immunity and nonspecific immune functions. It was reported that anti-TNF-*α* therapy was effective for several weeks in AS. We have shown the elevated TNF-*α* in joints, the primary source of which is the activated macrophages in chronic inflammatory disease [20]. The activated macrophages combined with apparent enhancement of IL-6 and IL-8 suggest two stages in this inflammatory process: firstly, Th1 cells activate the secretion of IFN-*γ*, leading to low level secretion of TNF-*α* and secretion of other macrophage cytokines (e.g., IL-6, IL-8); in the second stage, TNF-*α*, in combination with T-cell-derived IFN-*γ*, activates macrophages and polymorphonuclear neutrophils (PMN) which are attracted to the site of inflammation (by IL-8, e.g.), thus leading to self-amplifying inflammation [21, 22]. In our study, allicin significantly inhibited the secretion of TNF-*α*, IL-6, and IL-8 in AS model mice, indicating that allicin could reduce inflammatory reaction of AS efficiently.

Although the pathogenesis of AS is still not fully clarified, numerous studies have shown that HLA-B27 gene is closely linked to the occurrence of AS [23, 24]. HLA-B27 not only tents to be misfolded [25] but also forms heavy chain dimers by disulfide interaction [26, 27]. Both events are related to the triggering of inflammatory responses, including the release of inflammatory factors, such as TNF-*α* [25–27]. In our study allicin inhibited the release of three inflammatory factors, meanwhile it also inhibited the expression of HLA-B2704 protein expression. Thus, we speculate that allicin might suppress inflammation via inhibiting HLA-B2704 protein expression, although the exact mechanism deserves further investigation. Moreover, whether allicin could affect the activation of NF-*κ*B and/or other signal pathways related to AS should be conducted further as well [28, 29].

To sum up, our present research provides evidence that allicin can significantly reduce TNF-*α*, IL-6, and IL-8 secretion and HLA-B2704 protein expression in HLA-B2704 transgenic AS model mice. These results indicate that allicin would be a potential therapeutic nutraceutical for AS treatment. It is of great importance to identify the direct protein target of allicin as well as further molecular mechanisms in the future.
